# Leveraging Telehealth to improve access to care: a qualitative evaluation of Veterans’ experience with the VA TeleSleep program

**DOI:** 10.1186/s12913-021-06080-5

**Published:** 2021-01-21

**Authors:** Francesca M. Nicosia, Bhavika Kaul, Annette M. Totten, Molly C. Silvestrini, Katherine Williams, Mary A. Whooley, Kathleen F. Sarmiento

**Affiliations:** 1 San Francisco VA Medical Center, 4150 Clement Street, 151-R, San Francisco, CA 94121 USA; 2 University of California, San Francisco, San Francisco, USA; 3 Oregon Health & Science University, Portland, USA; 4 VA Puget Sound Health Care System, Seattle, USA

**Keywords:** Telehealth, Sleep medicine, Patient experience, Veterans, Qualitative research

## Abstract

**Background:**

Obstructive sleep apnea is common among rural Veterans, however, access to diagnostic sleep testing, sleep specialists, and treatment devices is limited. To improve access to sleep care, the Veterans Health Administration (VA) implemented a national sleep telemedicine program. The TeleSleep program components included: 1) virtual clinical encounters; 2) home sleep apnea testing; and 3) web application for Veterans and providers to remotely monitor symptoms, sleep quality and use of positive airway pressure (PAP) therapy. This study aimed to identify factors impacting Veteran’s participation, satisfaction and experience with the TeleSleep program as part of a quality improvement initiative.

**Methods:**

Semi-structured interview questions elicited patient perspectives and preferences regarding accessing and engaging with TeleSleep care. Rapid qualitative and matrix analysis methods for health services research were used to organize and describe the qualitative data.

**Results:**

Thirty Veterans with obstructive sleep apnea (OSA) recruited from 6 VA telehealth “hubs” participated in interviews. Veterans reported positive experiences with sleep telemedicine, including improvements in sleep quality, other health conditions, and quality of life. Access to care improved as a result of decreased travel burden and ability of both clinicians and Veterans to remotely monitor and track personal sleep data. Overall experiences with telehealth technology were positive. Veterans indicated a strong preference for VA over non-VA community-based sleep care. Patient recommendations for change included improving scheduling, continuity and timeliness of communication, and the equipment refill process.

**Conclusions:**

The VA TeleSleep program improved patient experiences across multiple aspects of care including a reduction in travel burden, increased access to clinicians and remote monitoring, and patient-reported health and quality of life outcomes, though some communication and continuity challenges remain. Implementing telehealth services may also improve the experiences of patients served by other subspecialties or healthcare systems.

## Background

Obstructive sleep apnea (OSA) is a highly prevalent chronic medical condition among Veterans enrolled in the Veterans Health Administration (VA) [1]. Between 2013 and 2018, utilization of VA sleep services increased an average of 16% per year [2, 3]. In 2018 alone, over 1 million Veterans received sleep apnea care from VA [2], exceeding capacity of VA sleep programs and exacerbating long wait times. When left untreated, sleep apnea is associated with increased risk of depression and anxiety [4], neurocognitive disfunction [5, 6], cardiovascular and cerebrovascular morbidities [7–9], in addition to motor vehicle accidents and premature death [10, 11]. However, the majority of OSA cases are undiagnosed [12], highlighting the urgent need for improved access to testing and treatment [13].

Although access to diagnostic sleep testing, sleep specialists, and treatment devices (continuous positive airway pressure, CPAP) are limited in both the VA and community settings, access is particularly limited for patients living in rural areas. The VA Office of Rural Health TeleSleep program, which focuses on VA’s 2.8 million rural Veterans, aims to bring sleep care to rural Veterans, improving access to care which is often not readily available in rural communities [2, 13]. TeleSleep leverages hub-spoke models that use home sleep apnea testing (HSAT), synchronous video visits and a web-based application for remote patient monitoring and communication (Remote Veteran Apnea Management Platform, REVAMP).

As the largest integrated healthcare system in the United States, VA has been on the forefront of developing and utilizing telehealth to reach its geographically dispersed patient population. In addition to better access and reduced wait times, telehealth is associated with improved patient outcomes and satisfaction, increased patient-provider communication, and can be cost-effective [13–17]. Leaders in VA sleep medicine recognized the potential for telehealth to improve access to testing, diagnosis and long-term management of OSA [18, 19], capitalizing on the existing positive perception of Veterans that telehealth affords both convenience and decreased travel burden [13, 20]. However, it is not clear whether the TeleSleep program improves Veterans’ overall care experience as a specialty care service. Furthermore, Veteran input has never been incorporated into operational decisions for TeleSleep, such as whether the care delivery methods in a provider-designed sleep telehealth program actually meet the needs of rural Veterans. This qualitative study examined Veterans’ experiences and identified factors affecting participation in the program.

## Methods

### Setting

The TeleSleep program facilitates partnerships between robust sleep programs at VA Medical Centers (VAMCs; “hubs”) and community-based outpatient clinics (CBOCs) or other VAMCs (“spokes”). Hubs are large (typically urban) VA hospitals with subspecialty care providers on site. Spokes are small (typically rural) VA clinics without subspecialty care providers on site. The first 2 years of TeleSleep implementation (April 2017-Sept 2019) included 7 hubs (Boise ID, Philadelphia PA, Phoenix AZ, Pittsburgh PA, Portland OR, and San Francisco CA, and Spokane, WA) and 41 spokes. TeleSleep has three domains of services: (1) virtual encounters (video, telephone, secure messaging); (2) home sleep apnea testing; and (3) Remote Veterans Apnea Monitoring Platform (REVAMP), a web-application enabling Veterans and providers to conduct asynchronous care and remotely monitor positive airway pressure (PAP) therapy and response to treatment.

### Study design

To evaluate Veterans’ experiences with the TeleSleep program, three study team members (a medical anthropologist and two clinician-researchers with expertise in telehealth and sleep medicine) developed a semi-structured interview guide designed to elicit Veterans’ experiences across a range of domains associated with accessing and engaging in sleep care (Appendix Table 2).

Sleep providers at 6 of the 7 hubs obtained patients’ permission to be contacted by the evaluation team for a one-time telephone interview. After providing verbal consent, participants completed a semi-structured interview with a study team member trained in qualitative methods (FN, MCS). Interviews averaged 30 min and were audio recorded and transcribed. As this study was part of larger quality improvement initiative evaluating the national implementation of the TeleSleep program and intended to inform VA operations, it was exempt from IRB review in compliance with VA Handbook 1058.05.

We analyzed interviews using an analytic matrix template organized by defined domain, which is a qualitative approach developed for rapid health services research [21]. Following each interview, we first populated the matrix with relevant content summaries, then reviewed interview transcripts to refine information contained in the matrix and identify relevant participant quotations. To ensure reliability and consistency, at least two authors coded each transcript and compared their findings. Authors FN, AT, and BK independently reviewed transcripts and summaries to ensure validity. Summary documents created for each domain were shared with the larger evaluation team, which discussed the analysis and reconciled differences in interpretation by consensus.

## Results

Thirty Veterans with obstructive sleep apnea (OSA) completed interviews. Participants were predominantly male (80%), White (92%), and lived in rural locations (90%) calculated according to Rural Urban Commuting Area (RUCA) codes (Table 1) [22].

**Table 1:** Patient characteristics (*n* = 30)^a^

**Age**	
Mean (STD)	60.5 (12.7)
Minimum, Maximum	29.3, 90.1
**Sex**	
Male	24 (80%)
Female	6 (20%)
**Race and ethnicity**	
White	28 (93%)
Black	0
Hispanic	0
Asian/PI/AM IND	1 (3%)
Multiple	1 (3%)
**Education level**	
HS	3 (10%)
Some college	14 (47%)
Associate’s degree	5 (17%)
Bachelor’s degree	1 (3%)
Post grad	4 (13%)
Unknown/Missing	3 (10%)
**Marital status**	
Married	21 (70%)
Single	3 (10%)
Divorced	3 (10%)
Widowed	2 (7%)
Unknown/Missing	2 (7%)
**Rurality**	
Rural	27 (90%)
Urban	3 (10%)
**Community care/CHOICE**^b^**user for sleep testing**	
Yes	3 (10%)
No	27 (90%)

^a^One patient’s spouse completed the interview as proxy; the patient’s demographic data was included here

^b^Patient-Centered Community Care and Veterans Choice Program pay for Veterans to obtain care from non-VA providers

Overall, patients reported positive experiences with the TeleSleep program. To illustrate the range of experiences and factors affecting participation in sleep care, we present findings across the following domains: 1) impact on symptoms, health, and quality of life; 2) impact on access to care; 3) experiences with technology; 4) Veteran preferences for location of care (VA vs. non-VA); and 5) Veterans’ recommendations for program improvement.

## Impact of TeleSleep on health and quality of life

Most Veterans interviewed (24 of 30) described sleep problems as their primary health-related priority with significant negative impacts on health and overall quality of life (e.g., “it affects my whole life”). Fatigue also negatively impacted participants’ relationships with family and friends (e.g., “most days I just didn’t feel like going out and doing anything or seeing anybody because I was just so tired.”) and work performance or safety (e.g., “I drive [a] truck … if I fall asleep driving somebody is gonna die.”).

Veterans reported participation in the TeleSleep program had positive impacts, with responses ranging from it helped “a little bit” to “tremendously.” Patients reported improvements in the following areas: 1) sleep quality; 2) other health concerns (e.g., cardiac conditions, diabetes); and 3) quality of life (e.g., including family and social relationships, and work performance and safety). One patient explained:

“Before, when [I] was waking up three or four hours a night, my husband couldn’t sleep. And sometimes I’d wake up and I’d be up for like four hours straight and fall back asleep right before I had to get up for work. And since [starting sleep care], I am out like a light. Instantly. It’s nice. It’s been great.”

Although using PAP was initially challenging for some patients, many described how “there was definitely a big change” after initiating treatment. One patient described how it became routine:

“It took a little adjustment. Probably two, three weeks to get used to it. We started out with a nasal mask and that didn’t work out all that well, so we did the full face. And it took a little bit of time to get accustomed to it. But now it’s kind of hard to sleep without it. It’s just kind of one of those things you put it on and you feel like it’s a part of your regimen.”

Changes to interpersonal relationships due to improved sleep included having energy to socialize and increased intimacy with spouses. One patient who had slept separately from his wife for 49 years reported:

“But now with the CPAP machine, no foolin’ ... for the last year [we] have been in bed just about every night … 99% of the time we’re in bed together, snuggling and I mean it’s like, this is wonderful.”

Other patients added that sleep care reduced their fatigue while at work. For example, a Veteran who worked as a maintenance director said,

“I just could see myself getting tired. Nine, ten o’clock in the morning after getting what I thought was a good night’s sleep …. I’d come home at lunch and take a power nap. ….but it seems like lately since I’ve been using the CPAP machine I don’t get as tired as frequently.”

Although most reports were positive, a few said it was “too soon to tell” because they had only recently seen a TeleSleep provider when interviewed and had not yet received test results or were waiting to receive PAP machine.

## Impact of TeleSleep technology on access to care

### Technology removes distance and travel barriers

Veterans overwhelmingly reported that telehealth facilitated access to sleep care because it alleviated the dual barriers of distance and travel time. As one patient explained:

“I live where it’s very, very rural. There’s a lot of lakes and hills and valleys. So for me to get to the local clinic, it’s a good 40 to 45-minute drive from where I live. So it’s not an easy day when I have to go there and back.”

In addition to travel and associated cost, some explained how health conditions presented additional barriers to accessing in-person care:

“I can’t afford it number one, and number two is an oxygen logistical problem because I’m on oxygen 24/7. I can’t take their VA transit [or] public transit because of the oxygen I have to carry. So I have to take my own vehicle and I can’t drive because of my sleep problem. So my brother-in-law has to take time to take me down there and we have to spend the night, and it is just a hardship.”

Notably, most rural participants indicated they would choose video visits over traveling to see sleep providers in person, explaining that miles and travel time ranged from inconvenient to almost impossible. Others expressed that video technology is a “wonderful innovation” enabling more frequent contact with sleep providers.

However, travel distance was not universally a barrier. A few participants, particularly those who were retired or did not rely on others for transportation, commented on “making a day out of it” by scheduling multiple appointments, social outings or errands. One Veteran explained:

“It’s an afternoon out. …It’s an hour or so drive there. I do my appointment with them. And then when I leave, there’s a bigger shopping center and supermarket than I have right here in the town area. I drop in there, I check that out. There’s a little Italian deli [where] I stop and then I go home.”

### Remote monitoring and access to personal sleep data

Remote monitoring of PAP devices is a core feature of TeleSleep. Data transmitted includes days and duration of device use, mask leak, and residual number of breathing events while on therapy. Prior to remote monitoring, Veterans received little feedback on their PAP compliance. Veterans perceived remote monitoring as either positive or neutral, with no negative feedback or privacy concerns. In fact, some Veterans explained how they found comfort in knowing providers can identify issues (e.g., “It’s helpful for the doctors.”). Others expressed feeling cared for and “comforted” knowing providers can monitor and address sleep issues. As one patient explained: “Oh, I think it’s great. I have no problems with [remote monitoring] at all. I need to be taken care of. I can’t do it on my own. I don’t know what to look for.”

Accessing their own sleep data was another positive aspect of the TeleSleep program’s technology platform. Specifically, Veterans commented on how the REVAMP web-application enabled them to monitor and improve their sleep quality. One participant who described himself as “competitive” explained:

“So if it gives you a score on how well you sleep, how long you have it on, and things like that. I would aspire to do better . . . And now I’m just about tapping out at a hundred every night. So it’s a good thing.”

Another commented on how data on the frequency of waking episodes provided insight, enabling them to adjust their sleep schedule in response:

“I like the knowledge, so that I knew after a week you can see how many times I get up in the middle of the night, how long I’m in bed tossing and turning and then the knowledge of going to bed later, so that I am tired and fall asleep.”

One patient commented on how their provider was able to adjust the their PAP settings remotely: “I have called [my provider] before and I said, ‘Do I need more humidity, or do I need this because my nose was dry?’ a couple times. But she adjusted it remotely and hacked it.”

### Experience with video technology

Overall, Veterans provided positive feedback about their experience seeing sleep providers through video appointments and REVAMP’s secure messaging feature. Specifically, patients rated ease of use and quality of video connection across all platforms as satisfactory or excellent (e.g., “clear” and “perfect”). One person commented on the multiple options available to connect with their sleep provider on a personal device:

“If I use my iPhone it goes, everything is bing, bang, boom, zap it’s there. But I like to use the computer because it’s a little bigger. A little nice to look at a person that’s bigger than a dime.”

## Veteran preference for accessing sleep care: VA vs. community providers

The VA has a history of purchasing non-VA care from local community providers through a fee-for-service reimbursement model when care is either not available or cannot be provided in a timely manner within the VA [23]. Only some Veterans in this study had experience with community care for sleep services. Based on prior experience and knowledge of current options, they overwhelmingly preferred VA TeleSleep compared to outsourced care. Veterans’ reasons for preferring the VA included: 1) convenience and affordability; 2) Veteran-centered, quality care; 3) availability of home sleep apnea testing; 4) lack of follow up from community providers.

First, the convenience of receiving care at one location within an integrated health system was a common reason cited for preferring VA for sleep care. One Veteran explained:

“VA needs the results anyway, and it’s like I eliminate a third party. I go straight to the horse, you know? Instead of through the water trough I feel. And it works out better because the VA has all my records.”

The affordability of accessing sleep care through the VA was another reason Veterans preferred the VA over community care. One Veteran whose wife accessed community sleep care explained, “My wife has to pay for her CPAP stuff. And with the VA, [mine is] funded. We’re lower income so it’s like, ah, let’s do the VA.” Another remarked that the VA, “ended up being financially more beneficial for me. In other words, it was cheaper.” Billing mistakes were another reason why Veterans preferred VA:

“We had huge billing issues because they [community care] billed it incorrectly with the outside clinic and they came out and said we owed $8000 to them and I had to fight that in order for it to be billed correctly so that the VA would pay for it.”

Second, Veterans’ highlighted the quality of care received from the VA, including a smooth referral process from their primary care provider to the TeleSleep program, positive relationships with staff and providers, and feeling respected and supported as Veterans. Veterans described initiating sleep care as “wonderful,” “flawless,” and that “the entire process has been really easy to manage and everybody has been very helpful, professional along the way.”

A common theme was appreciation for care that was sensitive to their needs as Veterans. For example, one Veteran said,

“I don’t like to use outside the VA. . . it’s not really my favorite. Being a Vet, you know a lot of the staffing are Veterans themselves, so you have some common ground with them.”

Another Veteran highlighted the importance of established relationships and rapport: “I like going to the VA because I like my doctor, I like the tech there, I like the, all the personnel that works in the clinic and also in the CPAP supply area.”

Third, almost half of Veterans interviewed had completed home sleep testing – a service offered through the VA to increase access. These Veterans described their experience with home testing positively, with few equipment or connectivity issues. Veterans who had previously completed in-laboratory testing reported that they preferred home testing because of difficulty sleeping in a lab. As one participant said, “It [in-lab testing] was more like an awake study, it was hard to fall asleep.” Another explained:

“I gotta drive three and a half hours up there, in the day. And then I’m supposed to calm down and sleep with all of these wires on me, and then as soon as they’re done at like 7 o’clock in the morning you’re just, you’re sent home. And so now I’m exhausted and I gotta drive three and a half hours home.”

Home testing eliminated the dual challenges of traveling to and falling asleep in a lab setting, with many describing the process as “simple” and “self-explanatory.”

Fourth, lack of follow-up from community providers was a contributing factor to Veterans preference for VA care. A common refrain from those who underwent outside testing was not receiving results: “I really didn’t get any results from the [testing] that was done through the [community] hospital.” Lack of follow-up from community providers was compounded by poor care coordination with VA providers. One Veteran explained how this resulted in him purchasing his own CPAP:

“The VA put me through CHOICE [outsourced care], CHOICE sent me to the guy. I never got a machine. Because it just went around and around and around. And finally, at that point I bought my own machine. Set it up for myself and took care of business. Until finally months later somebody called me up from the VA and I finally got them to give me another machine, but I already bought one.”

A minority of patients either expressed no preference or preferred the most convenient and closest option.

## Recommended changes: equipment and communication

Veterans identified three key areas for improvement: (1) appointment scheduling; (2) continuity and timeliness of communication; and (3) the equipment refill process. In general, Veterans stated that although their overall experiences with the program were positive, systems issues related to follow-up care needed improvement.

Veterans described challenges scheduling appointments. For example, one Veteran said, “The only issue I’ve had is with trying to make appointments and getting hold of a real person on the phone.” Others asked for quicker call backs and more streamlined scheduling processes.

Veterans also recommended improving communication to facilitate continuity and coordination of care. Specifically, some suggested having a single point of triage such as a VA telephone number that Veterans could call “to be able to pose questions” related to their sleep care.

Veterans reported challenges with supply refills and obtaining properly fitting masks. One Veteran explained that, “if your sleep apnea CPAP machine breaks, it’s really hard to get another one. It took me months of diddling around trying to get it.” The process for fitting and getting replacement parts and supplies was cited as an important target for program improvement.

## Discussion

In this qualitative evaluation of Veteran experience with the national VA TeleSleep program, Veterans reported high levels of satisfaction across multiple domains. Since implementation in 2017, the TeleSleep initiative has facilitated an increase in the number of Veterans diagnosed with OSA and in the number of encounters for sleep related disorders [3]. This study provides insight into Veterans’ experiences with TeleSleep and identifies factors affecting participation.

Veterans who engaged in TeleSleep care reported that decreased travel burden and increased convenience resulted in greater access to care. Convenience is a recognized advantage of telemedicine, particularly for rural Veterans who experience distance-related travel burden as a primary barrier to care [16, 20, 24]. However, not all Veterans in our study experienced distance as an insurmountable barrier to care. This echoes previous research among rural Veterans that found the degree to which distance is a barrier is relative to patients’ health status and resources, and the complexity and urgency of services [20].

The positive impact of TeleSleep care on patients’ health and quality of life was notable. Patient-reported outcomes ranged from improved sleep quality and other health-related issues to substantial improvements in quality of life. These patient perspectives support previous research demonstrating that sleep telemedicine can improve clinical outcomes [13]. In addition to health outcomes, our participants reported improvements among interpersonal and work-related aspects of their lives, important factors affecting overall quality of life.

Veterans in this study reported high levels of satisfaction and ease of use with digital platforms used to access care. Although telehealth has been shown to facilitate access to care [25], previous research has reported mixed results regarding patient satisfaction with telemedicine versus in-person visits [13, 14, 16, 26–28]. Our findings add to evidence that the quality of sleep telemedicine visits can be comparable to in-person care [13]. This is particularly noteworthy because the median age of patients in our study was 60.5, suggesting that older Veterans are increasingly comfortable with the use of technology for health care.

We also found overall positive attitudes toward remote monitoring of personal sleep data, an essential component of sleep telemedicine. Not only were Veterans’ accepting of remote monitoring, but some also found it “comforting” and an indication that they were being taken care of by sleep providers. Veterans in our study also appreciated the ability to access their sleep data through online monitoring platforms. Although some studies have identified lack of trust in VA and engagement issues as barriers to care, our findings regarding acceptability of remote monitoring suggest the potential for this platform to increase patient engagement in sleep care [29, 30].

Veterans in our study overwhelmingly preferred VA for their sleep care needs. The convenience of an integrated health care system and affordability of VA services were important factors shaping preference. Previous research has shown increased access to sleep testing through both home testing and community-care, particularly among rural Veterans [3]. Our findings provide insight into Veteran preferences for location of care, specifically, the availability of home testing as another reason for preferring VA over community care. In many rural areas, lack of specialists in the community is a known barrier to care [31]. Among patients who did access community care for testing, lack of follow up and poor continuity of care was an important factor influencing preference for VA, previously identified as a factor influencing satisfaction with outsourced care among both patients and providers [32, 33]. Our findings further reinforce the need to improve coordination between VA and the community to mitigate potential satisfaction and patient safety issues [3, 32].

### Limitations

Our interviews were limited to a convenience sample of patients from the ORH TeleSleep program and our findings may not be generalizable to non-Veteran patients or to Veterans receiving care at other VA facilities. Our results may have been affected by selection bias, as the perspectives of patients who chose to be interviewed could differ in important ways from those who declined. The experiences of younger Veterans were less well represented in our findings. Finally, our single-interview methodology may not have captured changes in patients’ perspectives and experiences after continued engagement with the TeleSleep program.

## Conclusions

The VA TeleSleep program facilitated improved patient experiences including: sleep quality, other health concerns, and quality of life; removes distance and travel barriers; and facilitates access to care for Veterans living in rural areas. Other VA specialty care services as well as other healthcare systems can learn from the experiences of patients served by the TeleSleep program. The positive experiences of patients with the program reassured program leaders and clinicians that the comprehensive shift in all VA sleep care to telehealth during the recent COVID-19 pandemic would facilitate improved access to care. Future work, informed in part by this qualitative evaluation, will focus on incorporating patient-driven self-scheduling and self-initiated supply refills, two initiatives developed in partnership with VHA’s Telehealth Service and Prosthetics and Logistics Offices, respectively. Understaffing of VA sleep programs, identified by Veterans as “timeliness of communication,” will remain a challenge. This specific barrier to care will continue to spark TeleSleep’s innovation, working toward new shared resource models and potential VA-Community collaborations.

## Data Availability

The datasets used and/or analysed during the current study are available from the corresponding author on reasonable request.

## Appendix

**Table 2:** Sample interview guide questions

**Experience with sleep problems**	
• What kind of problems with sleep have you been experiencing?	
• Compared to other health concerns, how important are your sleep problems to address? Why?	
• How has sleep disruption impacted your overall health and quality of life? Family and social relationships? Work responsibilities? Other activities?	
**Initiation and referral process**	
• What were the reasons why you decided to see a sleep specialist?	
• Tell me about the referral process for seeing the sleep specialist. What worked well and didn’t work well for you?	
• Had you been referred for an evaluation in the past? Did you engage in care at that time? Why or why not?	
**Overall experience with TeleSleep**	
• What do you like about TeleSleep visits? What don’t you like?	
• Aside from your sleep medicine physician, what other types of providers have you seen through Sleep care?	
• How did working with Sleep specialists change how you manage your sleep problems?	
• How did these changes impact your sleep quality and your day-to-day life?	
**Technology**	
• Which of the following methods have you used:	
○ Video chat visits at the clinic	
○ Video chat visits at home	
○ Email (secure messaging)	
○ Internet (REVAMP web app)	
• How easy or difficulty is it to use video, email, and/or internet.	
• How satisfied are you with the quality of the video connection?	
**Experience with sleep-apnea testing**	
• Have you participated in testing for sleep apnea/sleep problems? If yes: Did you do this testing at home or did you stay overnight for testing in a sleep lab?	
• If at home: Tell me about your experience using the testing equipment at home. What worked well about home testing? What didn’t work?	
○ How well does it work to connect the testing equipment where you live? Is there adequate cell coverage in your area to transmit data from the testing equipment?	
• If at lab: Tell me about your experience going to the lab for testing. What did you like? What didn’t you like?	
**Long-term management**	
• Do you use a CPAP machine for remote monitoring?	
○ Do you feel you received enough information on how to operate your CPAP machine? Why/why not?	
• Your CPAP device can send data about the status of your sleep apnea to your provider. How do you feel about this remote monitoring? (e.g., Has it helped you to get care more easily? Do you feel reassured someone is monitoring your progress with therapy? Is it invasive?)	
• Have you had any issues have with the fit or other aspects of your mask or other equipment? If so, tell me more.	
• How easy/hard has it been to contact your sleep providers when you need a follow up visit? Do you know who they are? When you want get in touch with them, how do you do that?	
**Knowledge of and experience with community care**	
• Aside from the VA, have you gotten sleep care from in community sleep specialists?	
• How far would you have to travel to get to a VA location to reive sleep care? How far would you have to travel to a community location to receive sleep care?	
• Would you have preferred to receive Sleep Care within the VA or community setting? Why or why not?	
**Recommended changes**	
• What would you change about VA Sleep care to improve your experience?	

## Abbreviations

CBOC: VA Community-based outpatient clinic
CPAP: Continuous positive airway pressure
HSAT: Home sleep apnea testing
ORH: VA Office of Rural Health
OSA: Obstructive sleep apnea
PAP: Positive airway pressure
REVAMP: Remote Veteran Apnea Management Platform
RUCA: Rural Urban Commuting Area
VA: Veterans Health Administration
VAMC: VA Medical Center

## References

[1] Yoon J (2011). Recent trends in veterans affairs chronic condition spending. Popul Health Manag.

[2] Sarmiento KF (2019). National expansion of sleep telemedicine for veterans: the TeleSleep program. J Clin Sleep Med.

[3] Weaver FM (2020). Comparing VA and community-based care: trends in sleep studies following the veterans choice act. J Gen Intern Med.

[4] Garbarino S (2020). Association of anxiety and depression in obstructive sleep apnea patients: a systematic review and meta-analysis. Behav Sleep Med.

[5] Lal C, Strange C, Bachman D (2012). Neurocognitive impairment in obstructive sleep apnea. Chest.

[6] Olaithe M (2018). Cognitive deficits in obstructive sleep apnea: insights from a meta-review and comparison with deficits observed in COPD, insomnia, and sleep deprivation. Sleep Med Rev.

[7] Marin JM (2005). Long-term cardiovascular outcomes in men with obstructive sleep apnoea-hypopnoea with or without treatment with continuous positive airway pressure: an observational study. Lancet.

[8] Shahar E (2001). Sleep-disordered breathing and cardiovascular disease: cross-sectional results of the sleep heart health study. Am J Respir Crit Care Med.

[9] Yaggi HK (2005). Obstructive sleep apnea as a risk factor for stroke and death. N Engl J Med.

[10] Tregear S (2009). Obstructive sleep apnea and risk of motor vehicle crash: systematic review and meta-analysis. J Clin Sleep Med.

[11] Leger D (2012). Impact of sleep apnea on economics. Sleep Med Rev.

[12] Kapur V (2002). Underdiagnosis of sleep apnea syndrome in U.S. communities. Sleep Breath.

[13] Fields BG (2016). Remote ambulatory management of veterans with obstructive sleep apnea. Sleep.

[14] Kruse CS (2017). Telehealth and patient satisfaction: a systematic review and narrative analysis. BMJ Open.

[15] Dellifraine JL, Dansky KH (2008). Home-based telehealth: a review and meta-analysis. J Telemed Telecare.

[16] Polinski JM (2016). Patients’ satisfaction with and preference for Telehealth visits. J Gen Intern Med.

[17] Hill RD (2010). Review of veterans health administration telemedicine interventions. Am J Manag Care.

[18] Singh J (2015). American Academy of Sleep Medicine (AASM) position paper for the use of telemedicine for the diagnosis and treatment of sleep disorders. J Clin Sleep Med.

[19] Colvin L (2019). Telehealth: helping solve a problem we created. J Clin Sleep Med.

[20] Buzza C (2011). Distance is relative: unpacking a principal barrier in rural healthcare. J Gen Intern Med.

[21] Hamilton AB (2013). Qualitative methods in rapid turn-around health services research. Health services research & development cyberseminar.

[22] Service, U.S.D.o.A.E.R. Rural-urban commuting area codes. 2019 [cited 2020 April 9]; Available from: https://www.ers.usda.gov/data-products/rural-urban-commuting-area-codes.aspx.

[23] Kaul B, H.D., Hickock A, Smith C, Niederhausen M, Totten A, Whooley M, Sarmiento K., Does community outsourcing improve access to care for Veterans with obstructive sleep apnea? Med Care. (under review). 10.1097/MLR.0000000000001472 PMC7899214 33290324

[24] He K (2017). Veteran preferences regarding wireless management of positive airway pressure for obstructive sleep apnea at a tertiary health-care system. Respir Care.

[25] Schooley BL (2010). Rural veteran access to healthcare services: investigating the role of information and communication technologies in overcoming spatial barriers. Perspect Health Inf Manag.

[26] Ackerman SL, Gleason N, Shipman SA (2020). Comparing patients’ experiences with electronic and traditional consultation: results from a multisite survey. J Gen Intern Med.

[27] Slightam C (2020). Patient perceptions of video visits using veterans affairs Telehealth tablets: survey study. J Med Internet Res.

[28] Gordon HS (2020). “I’m not feeling like I’m part of the conversation” Patients’ perspectives on communicating in clinical video Telehealth visits. J Gen Intern Med.

[29] Fischer EP (2016). Overcoming barriers to sustained engagement in mental health care: perspectives of rural veterans and providers. J Rural Health.

[30] Koenig CJ (2016). Pre-implementation strategies to adapt and implement a veteran peer coaching intervention to improve mental health treatment engagement among rural veterans. J Rural Health.

[31] Doyle JM, Streeter RA (2017). Veterans’ location in health professional shortage areas: implications for access to care and workforce supply. Health Serv Res.

[32] Schlosser J (2020). VA-community dual care: veteran and clinician perspectives. J Community Health.

[33] Nevedal AL (2019). A qualitative study of primary care providers’ experiences with the veterans choice program. J Gen Intern Med.

